# Chronic treatment with krill powder reduces plasma triglyceride and anandamide levels in mildly obese men

**DOI:** 10.1186/1476-511X-12-78

**Published:** 2013-05-27

**Authors:** Kjetil Berge, Fabiana Piscitelli, Nils Hoem, Cristoforo Silvestri, Ingo Meyer, Sebastiano Banni, Vincenzo Di Marzo

**Affiliations:** 1 Aker Biomarine ASA, Fjordallèen 16, NO-0115 Oslo, Norway; 2 Endocannabinoid Research Group, Institute of Biomolecular Chemistry, Consiglio Nazionale delle Ricerche, Via Campi Flegrei 34, Comprensorio Olivetti, 80078 Pozzuoli, (NA), Italy; 3 Momentum Pharma Services GmbH, Kieler Strasse 99-105, 22769 Hamburg, Germany; 4 Dipartimento di Scienze Biomediche, Università di Cagliari, Cittadella Universitaria, 09042 Monserrato, (CA), Italy; 5 Nutrisearch s.r.l., Edificio 5 A1 Parco scientifico e tecnologico Polaris, 09010 Pula, Italy

**Keywords:** Anandamide, *N*-acylethanolamines, Endocannabinoid, CB1 receptor, Obesity, n-3 polyunsaturated fatty acids, Krill oil, Fish oil

## Abstract

We have previously shown that treatment of Zucker rats and mice with diet-induced obesity with dietary docosahexaenoic (DHA) and eicosapentaenoic (EPA) acids in the form of krill oil reduces peripheral levels of endocannabinoids, ectopic fat formation and hyperglycemia. We reported that such treatment reduces plasma endocannabinoid levels also in overweight and obese human individuals, in whom high triglycerides may correlate with high circulating endocannabinoid levels. In this study, we report the effects of krill powder, which contains proteins (34%) in addition to krill oil (61.8%), on these two parameters. We submitted 11 obese men (average BMI of 32.3 kg/m^2^, age of 42.6 years and plasma triglycerides of 192.5 ± 96.3 mg/dl) to a 24 week dietary supplementation with krill powder (4 g/day *per os*) and measured anthropometric and metabolic parameters, as well as blood endocannabinoid (anandamide and 2-arachidonoylglycerol) and esterified DHA and EPA levels. Six subjects were included as control subjects and not given any supplements. The treatment produced, after 12 and 24 weeks, a significant increase in DHA and EPA in total plasma, a 59 and 84% decrease in anandamide plasma levels, and a 22.5 and 20.6% decrease in triglyceride levels, respectively. There was also a significant decrease in waist/hip ratio and visceral fat/skeletal muscle mass ratio at 24 weeks, but no change in body weight. These data confirm that dietary krill powder reduces peripheral endocannabinoid overactivity in obese subjects, and might ameliorate some parameters of the metabolic syndrome.

## Introduction

Endocannabinoids are endogenous arachidonic acid (AA)- and phospholipid (PL)-derived mediators that activate the G-protein-coupled receptors for Δ^9^-tetrahydrocannabinol, known as cannabinoid receptors of type-1 (CB1) and −2 (CB2) [1]. Overactivation of CB1 receptors by either of the two most studied endocannabinoids, i.e. *N*-arachidonoyl-ethanolamine (anandamide, AEA) and 2-arachidonoylglycerol (2-AG), contributes to metabolic dysfunctions in both obese rodents and humans [1]. Accordingly, plasma AEA and 2-AG levels, which reflect at least in part a spillover from peripheral organs involved in the control of lipid and glucose metabolism, are elevated in obese subjects [2-4]. Plasma AEA levels are sensitive to changes in the nutritional status, decreased after a meal in lean and obese humans and reduced by insulin infusion [2-4]. Plasma 2-AG levels, instead, positively correlate with intra-abdominal obesity, plasma triglyceride and markers of insulin resistance, and negatively with HDL-cholesterol [5,6]. Weight loss in intra-abdominally obese men is accompanied by reduced AEA and 2-AG levels, and the decrease of the latter correlates with weight loss-induced amelioration of high triglycerides, insulin resistance and HDL-cholesterol levels [7]. In mice, genetic deletion of the enzymes catalyzing endocannabinoid hydrolysis, i.e. fatty acid amide hydrolase and monoacylglycerol lipase, is followed by elevation of AEA and 2-AG levels, respectively. However, only deletion of fatty acid amide hydrolase is accompanied by increased body weight, and this phenotype is worsened by the occurrence of increased adipose tissue and triglyceride content in plasma, liver, skeletal muscle and adipose tissue following a high fat diet (HFD) [8-10].

Whilst acute or chronic elevation of peripheral endocannabinoid levels is accompanied by, and may contribute to, metabolic disorders in obese animals and human subjects, counteraction of CB1 activity with selective inverse agonists or antagonists reduces obesity and several ensuing metabolic disorders and counteracts the dysmetabolic effects of a HFD in rodents [11]. Also inhibition of the biosynthesis of endocannabinoids, namely 2-AG, can reduce food intake and body weight in rodents fed with a HFD [12]. Recently, we have shown that yet another strategy to reduce peripheral endocannabinoid overactivity in obese rodents and overweight/obese humans consists of the chronic supplementation with dietary docosahexaenoic (DHA) and eicosapentaenoic (EPA) acids in the form of krill oil [11,13-16]. In the case of Zucker rats [15] and mouse made obese with a HFD [16], this strategy was accompanied by a reduction of liver and heart ectopic triglyceride levels or of HFD-induced hyperglycemia, respectively. These effects were paralleled by the expected reduction of AA esterified into PLs, and by the finding of lower levels of AA-containing, direct endocannabinoid biosynthetic precursors in these tissues. Also in overweight/obese subjects the inhibitory effect of krill oil on endocannabinoid levels was correlated to a reduction of the n-6/n-3 long chain polyunsaturated fatty acid (LCPUFA) ratio in plasma PLs [13]. Alvheim and colleagues [17] recently showed that dietary linoleic acid (LA), which is abundant in several western-type diets, increases AA levels esterified into PLs, and, subsequently, 2-AG and AEA levels in the liver, and favors the development of diet-induced obesity in mice in a manner prevented by dietary EPA and DHA from fish oil, which reduced the AA-PL pool and normalized endocannabinoid tissue levels. These data indicate that diets rich in n-3 LCPUFA, such as those provided by fish or krill oil and krill powder supplementation, might produce beneficial effects on the metabolic disorders ensuing from a western type-like diet via reduction of endocannabinoid biosynthesis in peripheral tissues.

This study was conducted in human obese individuals to assess at the same time the effect of a krill preparation on peripheral endocannabinoid levels and different parameters of the metabolic syndrome. Twelve obese men were submitted to a 24 week treatment with 4 g/day (*per os*) of krill powder, which differs from krill oil since it also contains proteins. We measured various anthropometric and metabolic parameters, as well as plasma AEA and 2-AG levels and the incorporation of AA, DHA and EPA in plasma PL, at baseline and after 12 and 24 weeks. We also included in the study six untreated age- and BMI-matched subjects. We report that dietary krill powder reduces circulating AEA, but not 2-AG levels, and at the same time produces a beneficial effect on high triglycerides.

## Materials and methods

### Study design

This was a single-centre, open label, pilot study to explore effects of krill powder on overweight subjects. The study was performed at Momentum Pharma Services GmbH (Hamburg, Germany). Twelve subjects were given 4 g krill powder daily as 500 mg gelatin capsules (active treatment group) for 24 weeks. The krill powder capsules were obtained from Aker BioMarine ASA (Oslo, Norway), and the composition of the krill powder is shown in Table 1. Six subjects were included as controls and received no study products. All subjects included in the study were males between 30 and 55 years, BMI 30–35 kg/m^2^, healthy and non-smoking. Subjects with a known history or presence of a significant cardiovascular disease, allergy towards crustaceans, bleeding disorders, disturbed absorption due to changes in gastro-intestinal tract, history of drug or alcohol abuse, contraindications of an MRI procedure, or with a concomitant administration of any medication which were known to be lipid modifying agents, were excluded from the study. Active treatment group had to oblige to restrictions which included restriction of fatty fish or seafood meals (salmon, mackerel, eel, halibut, trout, herring) to only once per week, but not within 48 hours before clinic visits, prohibition of functional food like cholesterol-reducing products and lipid supplements.

**Table 1:** Composition of krill powder

	**%**	**Content in**
	**in powder**	**4 g krill powder (mg)**
Proteins	34.0	1360
Total lipids	61.8	2472
Triglycerides	31.5	1260
Total PLs	25.4	1016
Total omega-3	13.7	548
Total omega-6	1.4	56
Saturated FAs	17.9	716
Total PUFAs	15.6	624
Total MUFAs	13.2	528
EPA	6.7	268
DHA	3.3	132

*DHA*: docosahexaenoic acid; *EPA*: eicosapentaenoic acid; *FA*: fatty acids; *MUFAs*: monounsaturated fatty acids; *PLs*: phospholipids; *PUFAs*: polyunsaturated fatty acids.

### Study assessments

At the screening visit, demographic data, medical history, diet, smoking and caffeine status was logged, and serology tests, urine drug screen and an alcohol breath test were performed. The screening was performed between 2 and 31 days before the treatment phase.

At all visits throughout the study (screening, week 0, 12, and 24), subjects had their body weight, waist-hip ratio and body fat percentage measured. MRI scans (both breath-hold and respiratory-triggered) were performed and fasting glucose and insulin levels were measured at week 0, 12 and 24. At all visits, blood for the assessment of esterified fatty acids, lipids in serum (total cholesterol, LDL-cholesterol, HDL-cholesterol, triglycerides) and endocannabinoids were collected from fasted subjects. Subjects in the control group were only assessed for fasting endocannabinoid levels at week 0, 12 and 24.

Safety assessments were performed at screening and all visits in the study. Safety assessments included inquiry about adverse events and concomitant medication at all clinic visits and at phone calls (between all visits during treatment), measurement of vital signs (blood pressure, pulse rate, body temperature), physical examination, the recording of a 12-lead ECG, and a standard clinical laboratory assessment (urinalysis, haematology, clinical chemistry).

### Ethics

The study was conducted in compliance with the ethical principles that have their origin in the Declaration of Helsinki and that are consistent with Good Clinical Practice (GCP), as described in Declaration of Helsinki and ICH Harmonized Tripartite Guidelines for Good Clinical Practice. The final protocol and the Informed Consent Form (ICF) were approved by the Ethics Committee of the Ärztekammer (Medical Chamber), Hamburg, Germany.

The Investigator ensured that subjects were given full and adequate oral and written information about the nature, purpose, possible risks, and benefits (comprehensive medical screening) of the study. Subjects were also notified that they were free to discontinue their participation in the study at any time. Subjects were given time for consideration and to have the opportunity to ask questions. The subject’s signed ICF was obtained before conducting any procedure related to the study.

### Body measurements

Subjects had their body weight, waist-hip ratio and body fat percentage measured at Screening, baseline and 12 and 24 weeks (±1 week). Subjects were measured barefoot in light underwear following manufacturer’s instructions. The body fat percentage was measured using BC-545 Segmental Body Composition Analyser (Tanita Europe GmbH, Sindelfingen, Germany). This device uses an 8 electrode segmental Bioelectrical Impedance Analysis (BIA) to monitor multiple components of overall health. Measurements included weight, body fat %, body water %, muscle mass, and visceral fat rating. Manufacturers’ equations were used to predict body fat %, body water %, muscle mass, and visceral fat rating.

### Magnetic Resonance Imaging (MRI)

Abdominal body fat was measured by using the Siemens Magnetom Avanto (76 × 18) Q-engine, SW B15 system. MRI scans (both breath-hold and respiratory-triggered) were performed at baseline and after 12 and 24 weeks of treatment at Radiologische Praxis Altona (Hamburg, Germany) by using the Dixon technique. The technique acquires two separate images with a modified spin echo pulse sequence. One is a conventional spin echo image with water and fat signals in-phase and the other is acquired with the readout gradient slightly shifted so that the water and fat signals are 180 degrees out-of-phase. From these two images, a water only image and a fat-only image could was generated, and the availability of both the water-only and fat-only images allowed direct image-based water and fat quantitation. The total volume of the target region was determined in cm^3^ as an outcome [18].

### Glucose, insulin and adiponectin measurements

For the fasting blood glucose and insulin assay, a 7.5 ml serum monovette was used after being left 30 min standing at room temperature and then centrifuged for 15 min at 2500xg at room temperature. The sample was divided in aliquots sent ambient to Bioscientia (Hamburg, Germany) for analysis of glucose and insulin.

### Fatty acid analysis

Plasma samples were thawed in fridge overnight, vortexed, centrifuged and pipetted into vials. Internal standard (triheptadecanoin) is added and samples are methylated with 3N MeOH HCl. FAMEs were extracted with hexane, then samples are neutralized with 3N KOH in water. After mixing and centrifuging the hexane phase is injected into the GC-FID.

Analysis was performed on a 7890A GC with a split/splitless injector, a 7683B automatic liquid sampler, and flame ionization detection (Agilent Technologies, Palo Alto, CA). Separations were performed on a SP-2380 (30 m × 0.25 mm i.d. × 0.25 μm film thickness) column from Supelco.

### Endocannabinoid analysis

Lipids were extracted from plasma (5 ml) and AEA and 2-AG pre-purified and quantified by isotope dilution- liquid chromatography-atmospheric pressure chemical ionization- mass spectrometry (LC-APCI-MS) as described previously [19-21]. Also the endogenous AEA congeners, palmitoylethanolamide (PEA) and oleoylethanolamide (OEA), were measured using the same methods, and as described previously [19]. Data were analyzed as the means of the percent changes from baseline (beginning of study) levels.

### Statistical analysis

For statistical testing, a paired T-test was used. The 12 and 24 week measurements were tested against baseline values. Significance levels were set to *P* < 0.05. All statistical tests were performed with JMP 10.0.2 (SAS Institute).

## Results

One subject in the active treatment group was excluded from the analysis, since he was lost to follow-up in the middle of the study. A number of 11 subjects in the active treatment group and all 6 subjects in the control group completed the study. There were no major protocol deviations or serious adverse events in the study, and the study product was well tolerated.

Dietary supplementation with 4 g/day (*per os*) of krill powder produced a significant increase in esterified DHA and EPA after both 12 and 24 weeks (*P* < 0.05) (Table 2). Therefore, percent wise, there was a reduction in the levels of esterified AA (as well as other fatty acids) relative to DHA and EPA (Table 2). Krill powder also produced: 1) a ~22.5 and 20.6% decrease in triglycerides levels after 12 and 24 weeks, respectively, although this was statistically significant (P < 0.05) only at 24 weeks (Table 3), and 2) a ~2.9% decrease in waist/hip ratio, also statistically significant at 24 weeks. No effect on any of the other metabolic or biochemical parameters was observed. Krill powder supplementation also produced a non-statistically significant, time-dependent trend towards decreased visceral fat, measured by the 3D three-point Dixon MRI, at both 12 and 24 weeks (Table 3). Interestingly, this trend became significant at 24 weeks if normalized by the percent change in skeletal muscle mass (Table 3). Eight subjects out of 11 showed slight reductions in the measured abdominal body fat (not shown). Slight increases were noticed in mean values of total muscular mass as well as total water content after 24 weeks of active treatment. In addition, mean computed integral liver fat peak value, measured by the Proton liver SVS, also showed non-statistically significant reductions from baseline to week 24 (Table 3). At week 12, 4 subjects showed a decrease in the measured liver density, whereas 5 subjects showed a decrease in the measured liver density at week 24 (not shown). There was no effect on body weight at any time point.

**Table 2:** Effect of krill powder supplementation on plasma esterified fatty acid composition in obese men (N = 11) at baseline, the middle and end of the study

**Fatty acid**	**Baseline**	**Week 12**	**Week 24**
C12:0	0.10 ± 0.05	0.12 ± 0.07	0.12 ± 0.11
C14:0	1.25 ± 0.41	1.18 ± 0.41	1.19 ± 0.56
C15:0	0.20 ± 0.04	0.19 ± 0.04	0.20 ± 0.05
C16:0	23.27 ± 2.53	22.28 ± 2.41	22.78 ± 3.10
C16:1	2.49 ± 0.52	2.03 ± 0.59 **	2.19 ± 0.82
C18:0	6.56 ± 0.37	6.45 ± 0.46	6.42 ± 0.41
C18:1,t6-11	0.35 ± 0.13	0.47 ± 0.32	0.35 ± 0.14
C18:1,c9	23.10 ± 2.54	22.06 ± 2.05	21.54 ± 2.21 **
C18:1,c11	1.56 ± 0.29	1.52 ± 0.21	1.56 ± 0.25
C18:2,n-6	23.11 ± 3.78	25.25 ± 4.06 **	24.37 ± 4.99
C20:0	0.25 ± 0.05	0.25 ± 0.04	0.25 ± 0.05
C18:3,n-6	0.45 ± 0.18	0.37 ± 0.09	0.35 ± 0.09
C18:3,n-3	0.73 ± 0.30	0.75 ± 0.26	0.73 ± 0.19
C20:1,n-9	0.15 ± 0.05	0.15 ± 0.04	0.16 ± 0.04
C20:2,n-6	0.19 ± 0.04	0.18 ± 0.03	0.17 ± 0.04
C22:0	0.56 ± 0.13	0.60 ± 0.14	0.60 ± 0.16
C20:3,n-6	1.51 ± 0.21	1.33 ± 0.17 *	1.38 ± 0.15 *
C20:4,n-6 (AA)	4.85 ± 1.15	5.00 ± 0.78	5.30 ± 1.13
C20:4,n-3	0.10 ± 0.04	0.10 ± 0.03	0.09 ± 0.03
C20:5,n-3 (EPA)	0.75 ± 0.24	1.24 ± 0.20 ***	1.15 ± 0.43 ***
C24:0	0.54 ± 0.15	0.54 ± 0.16	0.54 ± 0.16
C24:1	1.00 ± 0.30	1.02 ± 0.19	1.04 ± 0.29
C22:5,n-3	0.47 ± 0.08	0.53 ± 0.08 *	0.52 ± 0.08 *
C22:6,n-3 (DHA)	1.37 ± 0.32	1.67 ± 0.37 *	1.72 ± 0.28 ***

Data are expressed as mean g/100g FAME in plasma ± S.D. P-value was calculated at week 12 and 24 *vs.* baseline using a paired T-test. *, P < 0.05; **, P < 0.01; ***, P < 0.005 vs baseline.

**Table 3:** Biochemical and anthropometric measures of the volunteers (N = 11) at baseline, the middle and end of the study

**Parameter**	**Baseline**	**Week 12**	**Week 24**
Cholesterol (mg/dL)	210.3 ± 27.7	203.0 ± 39.5	206.3 ± 40.8
HDL (mg/dL)	45.1 ± 9.5	43.2 ± 10.5	43.6 ± 9.8
LDL (mg/dL)	139.1 ± 24.2	133.0 ± 28.6	137.9 ± 33.6
Triglycerides (mg/dL)	192.6 ± 96.3	149.3 ± 70.5	152.8 ± 96.2 *
VAT (L)	16.73 ± 2.55	16.29 ± 3.13	16.15 ± 2.42
Liver density (integral)	84.3 ± 79.2	79.2 ± 91.5	69.2 ± 74.6
Insulin (uU/ml)	15.5 ± 14.4	18.7 ± 11.9	17.4 ± 9.1
Glucose (mg/dL)	96.7 ± 13.6	99.6 ± 13.0	101.2 ± 18.4
Body fat (%)	28.4 ± 2.9	28.0 ± 3.1	27.9 ± 2.9
Weight (kg)	103.8 ± 12.6	104.0 ± 13.7	104.0 ± 13.4
Muscular mass (kg)	70.6 ± 8.9	71.1 ± 9.3	71.3 ± 9.1
Water (%)	50.8 ± 2.7	51.1 ± 2.8	51.3 ± 2.5
Waist/hip ratio	1.01 ± 0.04	0.99 ± 0.07	0.98 ± 0.05 *
VAT/muscular mass ratio	0.24 ± 0.04	0.23 ± 0.04	0.23 ± 0.03 *
Body fat/muscular mass ratio	0.41 ± 0.08	0.40 ± 0.08	0.40 ± 0.07

*VAT*: visceral adipose tissue. P-value was calculated at week 12 and 24 *vs.* baseline using a paired T-test. * P-value < 0.05 vs baseline.

**Figure 1:**
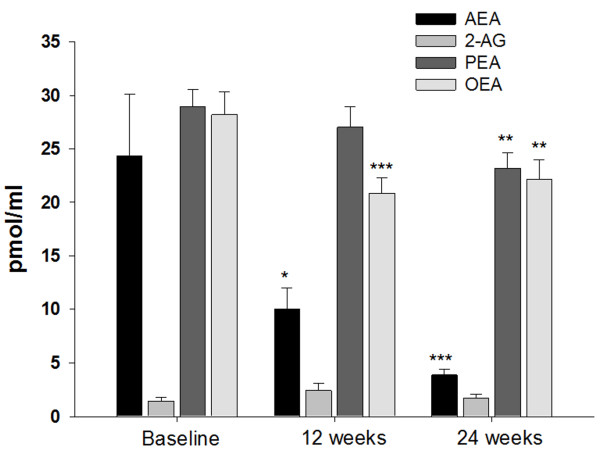
**Effect of 4 g daily krill powder supplementation on plasma anandamide (AEA), 2-arachidonoyl-glycerol (2-AG), palmitoylethanolamide (PEA) and oleoylethanolamide (OEA) levels in obese men after 12 and 24 weeks (N = 11).** *, P < 0.05; **, P ≤ 0.01; ***, P < 0.005 vs baseline (paired t-test).

Concomitantly to these changes, krill powder supplementation produced a 59 and 84% statistically significant decrease in AEA plasma levels at 12 and 24 weeks, respectively (Figure 1). There was also a statistically significant, albeit smaller, decrease in PEA and OEA levels. The levels of 2-AG were not altered (Figure 1). No change in the levels of these compounds in the control group was observed at any time point (data not shown). No statistically significant correlation was found between endocannabinoid, OEA or PEA and triglyceride levels at any time.

## Discussion

We have reported here that dietary supplementation to obese men of DHA and EPA, in the form of krill powder, efficiently and persistently reduces high triglycerides independently from any overall effect on body weight, and at the same time lowers plasma AEA levels, i.e. a marker of obesity thought to contribute, via peripheral CB1 receptor overactivation, to obesity-associated dysmetabolism [1].

The effect of krill powder on high triglyceride levels appeared to be maximal already after 12 weeks of supplementation, and persisted until the end of the study, i.e. at 24 weeks. This suggests that this effect was of a relatively rapid onset, sustained and not merely due to subjects undergoing periodic medical control.

Unlike triglycerides, the effect on plasma levels of the endocannabinoid AEA was already significant after 12 weeks, but was maximal after 24 weeks of supplementation. Thus, possibly also due to the relatively low number of subjects recruited for this study, no correlation was found between plasma AEA and triglyceride levels, either before, in the middle or at the end of the dietary supplementation. Interestingly, previous studies showed that, of the two endocannabinoids, AEA and 2-AG, it is rather the plasma levels of the latter compound that, in overweight/obese individuals, directly correlate with plasma triglycerides, as well as with other anthropometric and metabolic parameters, such as visceral adipose tissue, insulin resistance and low HDL-cholesterol [5,6]. Furthermore, weight loss in abdominally obese men after one year of lifestyle (caloric restriction and exercise) intervention caused a reduction of the levels of both AEA and 2-AG, but only the latter: 1) correlated with weight loss-induced reduction in visceral adipose tissue, plasma triglycerides, insulin resistance and low HDL-cholesterol, and 2) through multivariate analyses was found to be an independent predictor of some of the observed reduction in plasma triglycerides [21].

Krill powder supplementation in this study: 1) reduced AEA, but not 2-AG, levels, this effect becoming maximal after the effect on triglyceride levels; and 2) produced only a trend towards the reduction of intra-abdominal fat. Therefore, it is tempting to speculate that krill oil-induced reduction of AEA levels is an effect that either occurs independently from the effect on triglycerides, or contributes, via reduced CB1 activation in peripheral organs, only to the sustained reduction of triglyceride levels. On the other hand, it was surprising to find that a reduction in plasma triglyceride levels (~20-22%) similar to the one (22.2%) previously shown [21] to be accompanied by reduced 2-AG levels, did not produce a similar effect in the present study, despite the fact that, as mentioned above, 2-AG is considered an independent predictor of at least part of plasma triglyceride levels in intra-abdominally obese men. It is possible that such correlation holds true only after weight loss, an effect not observed here, or following stronger reductions in intra-abdominal fat, or only in intra-abdominally obese men, as were the subjects of the previous study (who exhibited an average BMI of ~30 at the beginning of the study). Alternatively, it is also possible that the low number of obese men who underwent krill powder treatment (n = 11), a limitation of the present study, or the shorter duration of the present treatment as compared to the 1 year lifestyle intervention used in the previous study [20], prevented us from observing a reduction in 2-AG levels. However, it must be pointed out that a statistically significant, albeit small, effect on waist/hip ratio and visceral fat (if normalized to skeletal muscle mass) was observed here with krill powder. The former effect is in agreement with the previous finding of an association between high plasma AEA levels and waist circumference in mixed gender (predominantly female) non-obese and obese cohorts with average BMI of 21 and 42, respectively [3].

Whilst dietary supplementation of DHA and EPA in the form of fish oil had been already shown to reduce triglycerides in human subjects with signs of the metabolic syndrome [22-26], before the present report no such human data existed with regard to krill preparations. Importantly, in order to obtain a decrease of triglycerides similar to that found in the present study, higher doses of EPA plus DHA (3–4 g/day) in the form of fish oil are required [27]. It has been claimed that fish oil exerts its effect through PPARα activation in the liver by DHA, thus strongly increasing fatty acid β-oxidation and reducing hepatic triglyceride formation and release [28]. Krill oil and krill powder, however, differ from fish oil as they contain DHA and EPA mostly esterified to PLs rather than triglycerides. More than 80% of the EPA and DHA in krill oil/krill powder are associated with PLs, and the remaining is in triglyceride form [29]. Interestingly, both dietary krill oil and n-3 LCPUFAs supplemented as PLs were recently shown to produce stronger beneficial effects than fish oil or n-3 LCPUFAs supplemented as triglycerides, respectively, on ectopic triglyceride accumulation, inflammation and other metabolic dysfunctions in obese Zucker rats and DIO mice [15,30]. In these previous animal studies, krill oil or n-3 LCPUFAs supplemented as PLs were also found to be more effective at reducing endocannabinoid levels in peripheral organs, and, in Zucker rats, a stronger effect was found on AEA rather than 2-AG levels [15,29]. On the other hand, in a cohort of mostly women with average BMI of ~31, administered with dietary krill oil at a dose of 2.5 g/day, the endocannabinoid the plasma levels of which were reduced after 4 weeks treatment was 2-AG and not AEA. This effect was more efficacious than with an n-3 LCPUFA-equivalent amount of fish oil, which, in fact, did not produce any effect [13]. Therefore, it is possible that treatments with different formulations of krill oil, in different cohorts of subjects, and for a shorter duration, may produce different effects on plasma endocannabinoid levels. However, regardless of the type of formulation, protocol of administration and subjects or animal model of obesity undergoing the supplementation, the effect of krill oil on the peripheral levels of AEA and/or 2-AG is always accompanied by a reduction in the levels of esterified AA relative to EPA and DHA levels. This effect was observed also in the present study and we hypothesize that it may lead to an overall reduction of AEA biosynthetic precursor, *N*-arachidonoyl-phosphatidylethanolamine, which in turn would be responsible for the observed reduction in plasma AEA levels. Indeed, in the previous study in which n-3 LCPUFAs were supplemented as PLs to DIO mice [30], the reduction in white adipose tissue AEA levels was found to be accompanied by an equivalent elevation of the levels of its DHA- and EPA-derived congeners. Therefore, rather than, or in addition to, activation of PPARα, dietary EPA and DHA, especially in the form of krill preparations, might produce their effects on fasting triglycerides in obesity by re-equilibrating endocannabinoid levels and CB1 receptor tone. Interestingly, a decrease of the LCPUFA n-6/n-3 ratio in the plasma of hypercholesterolemic and overweight subjects, obtained with a different dietary intervention, was recently reported to be still accompanied by a 40% decrease in plasma AEA levels, but to be associated, instead, with a significant decrease of LDL-cholesterol [31]. Moreover, krill powder also contains astaxanthin which could theoretically cause biological effects, but the concentration is believed to be too low to be of any relevance (approximately 200 μg in 4 g krill powder).

It was interesting to find here how the levels of two AEA-related metabolites, OEA and PEA, changed in the same direction as AEA following dietary krill powder supplementation. These two compounds are inactive at cannabinoid receptors but can stimulate peroxisome proliferator-activated receptor-α and transient receptor potential vanilloid type-1 channels [19], two lipid-sensitive proteins which have been involved in the control of metabolism. In a previous study in human lean, obese and type 2 diabetes obese subjects, the plasma levels of AEA, OEA and PEA were found to correlate with each other [18], which is not surprising since these three compounds share similar, although not fully overlapping, biosynthetic and degrading routes and enzymes (see [18,19] and references cited therein). Therefore, we hypothesize that the small but significant reduction in the plasma levels of PEA and OEA might be due not only to the decrease of esterified palmitic and oleic acid relative to DHA and EPA, but also to alterations in the expression of their metabolic enzymes, as previously observed in osteoblasts [32], and that this might have contributed also to AEA, but not 2-AG, level reduction.

In conclusion, we have shown here that dietary supplementation with a krill powder persistently ameliorates high triglycerides without weight loss in obese men, to an extent similar to that observed following lifestyle-induced weight loss in a previous study in dyslipidemic overweight/obese men [21], and in a shorter period of time (12–24 weeks *vs*. one year). The understanding of whether or not this metabolic effect of dietary DHA and EPA is due to its concomitant reduction of plasma AEA levels, i.e. a potential index of peripheral CB1 receptor activity in humans, and of its subsequent action on lipid metabolism and triglyceride clearance evidenced in rodents [9,10], will probably require further investigations with larger cohorts of subjects, varying doses of krill powder and several different time points. At any rate, given the ever increasingly established role of high triglycerides as an early determinant of insulin resistance, type 2 diabetes, atherosclerosis and cardiovascular risk in overweight and obese subjects, the present data suggest that dietary krill powder supplementation might represent a novel preventive strategy for these disorders.

## Competing interests

KB and NH are employees of Aker Biomarine, Norway.

## Authors’ contributions

KB, NH, IM, SB and VD designed the study. IM contributed to the performance of the trial. FP and CS performed plasma endocannabinoid analyses. KB, SB and VD wrote the paper. All authors read and approved the final manuscript.
